# Melatonin as preemptive analgesic for intraoperative pain

**DOI:** 10.6026/97320630019005

**Published:** 2023-01-31

**Authors:** Chanchal Katariya, Sankari Malaiappan

**Affiliations:** 1 Department of Periodontics, Saveetha Dental College, Saveetha Institute of Medical and Technical Sciences (SIMATS), Saveetha University, Chennai, India

**Keywords:** Melatonin, preemptive analgesic, intraoperative

## Abstract

Several anti-inflammatory and analgesic drugs have been used to reduce pain and discomfort during periodontal surgeries. This study evaluates the efficacy of using melatonin and ketorolac for pain prevention during open-flap debridement surgery. This prospective randomized controlled trial was performed in patients who presented with chronic periodontitis after non-surgical periodontal therapy, requiring flap surgery. Group 1: Flap surgery following non-surgical periodontal therapy after one month with no oral administration of analgesic. Group 2: Flap surgery following non-surgical periodontal therapy after one month with oral administration of Ketorolac 400mg one hour prior to the surgery. Group 3: Flap surgery following non-surgical periodontal therapy after one month with oral administration of 2 mg Melatonin one hour prior to the surgery. VAS and FLACC score along with blood pressure, SPO2 and heart rate. Statistical analysis was done using SPSS software. The intragroup comparisons (control-test drug) demonstrated that melatonin and ketorolac showed positive preemptive effect which compared to the control with mean differences significantly different from zero. However, when melatonin and ketorolac were compared there was no significant difference in postoperative pain among patients. The adoption of a preemptive medication protocol using either melatonin or ketorolac may be considered effective for pain and discomfort prevention during and post open-flap debridement surgeries. Melatonin showed similar effect to gold standard ketorolac in terms of its preemptive analgesic effect additional to having anti-inflammatory effect. Further studies are required to standardize the protocol for using melatonin as preemptive analgesic for dental surgical procedures.

## Background:

Pain is pretty ubiquitous in all medical situations, and its management is crucial in therapeutic and clinical practice. Pain and discomfort during treatment is one of the major problems faced by periodontists and patients during periodontal surgical procedures. It's a difficult and crucial problem to provide safe and effective analgesia for procedure pain. The ideal agent would be cheap, non-parentally administered, and offer effective, safe analgesia with rapid onset and offset. For surgical therapy in dentistry, various prophylactic analgesia strategies have been used. The following are the three primary factors in each of these protocols: drug selection, drug dose, and administration time. Morphine, its equivalents, non-steroidal anti-inflammatory medications (NSAIDs), and acetaminophen are some of the most widely used and effective pain relievers. However, the majority of these analgesics have significant adverse effects. NSAIDs are known to induce gastrointestinal damage, notably in the stomach and duodenum, which manifests clinically as ulcers and inflammation. [1] To overcome these disadvantages of analgesics, melatonin is considered to have good analgesic effects without any adverse effects. The pineal gland and several other organs, including the retina, bone marrow, and intestines, produce and emit melatonin (N-acetyl-5-methoxy-tryptamine), an indoleamine, in a circadian pattern. Additional pineal sites do not significantly increase melatonin production, and they also require particular stimuli in order to produce melatonin. [2] In 1958, melatonin was discovered and first described [3]. A variety of physiological processes are mediated by indoleamine binding to membrane receptors in all tissues. [4][5] Due to its lipophilic nature, melatonin can bind to some cytosolic proteins like kinase-C [6], calmodulin, and calreticulin [7] in the mitochondria of cells [8], allowing it to penetrate the subcellular compartment and reside in high concentrations there. The enzymes tryptophan-5-hydroxylase and 5-hydroxytryptophan decarboxylase, which successfully hydroxylate and decarboxylate tryptophan, respectively, assist the pinealocytes absorb free tryptophan from the blood and convert it into serotonin, which is then turned into melatonin. Serotonin is transformed to N-acetylserotonin throughout the night by the enzyme N-acetyltransferase, which is then methylated to create melatonin by the enzyme hydroxyindole-O-methyl transferase. Melatonin levels in all animals, including humans, peak in the middle of the night and drop to very low levels throughout the day. [9] Melatonin is referred to as the chemical manifestation of darkness because it is produced primarily at night. Melatonin is not regarded as a hormone because it is produced in various organs and has no effect on any particular organ. [10] Melatonin production declines when people get older. Melatonin may quickly traverse the blood-brain barrier, the placenta, and any other potential cellular obstructions because it has no morpho-physiological barriers. [11] Melatonin affects circadian rhythms [12-13], body temperature [14], sexual development [15] and the reproductive cycle, and the immune system. It also regulates body temperature. Melatonin functions as an antioxidant, an anti-inflammatory, and plays a crucial role in bone production and the decrease of bone resorption in the oral cavity. It also promotes other anti-oxidative enzymes. Melatonin exerts paracrine effects on adjacent cells. It controls interleukin-2 (IL- 2) and interferon-alpha secretion, acting as an immune-modulator and stimulates the production of type I collagen and bone formation. [16] Melatonin is released into the saliva after entering the blood. Only a minor part of the plasma's melatonin is free-floating, with the majority of it being attached to albumin. [6] The influence of melatonin as a preemptive analgesic for pain during periodontal flap surgery is not established. Therefore, it is of interest to probe the effect of pre-procedural analgesia on postoperative pain and interference in daily activities and to evaluate the effect of prophylactic analgesia on intraoperative pain and discomfort.

## Material and Methods:

This study was carried out in the Department of Periodontics and implantology, Saveetha Dental College (Chennai, Tamilnadu, India) and this study was approved by the Saveetha ethical committee and approval number- IHEC/SDC/PERIO-2001/22/578

## Trial design and randomization:

This is a randomized, controlled, single blinded, mono centric clinical trial. Simple Randomisation was done by asking the patient to pick a chit with the type of intervention written on it. The study was categorized in three phases:

[1] Enrollment

[2] Treatment

[3] Data evaluation and analysis

Enrollment included Evaluation of patient's eligibility, medical and dental history and full mouth periodontal charting.

## Participants:

## Exclusion criteria:

[1] Smoker's and tobacco users.

[2] Pregnant ladies and lactating women.

[3] Uncontrolled diabetes.

[4] Patient who has undergone periodontal therapy before 3 months.

[5] Patient on any antibiotic therapy.

[6] Patient on mood modulators or sedatives.

[7] Patient with history of any drug allergies.

## Inclusion criteria:

[1] Systemically healthy individuals.

[2] Aged between 18yrs - 65yrs.

[3] Untreated Stage II - Stage III periodontitis.

90 periodontitis patients from outpatient Department of Periodontology and Implantology, Saveetha Dental College and Hospitals were recruited for the study. Patients were enlightened about all the particulars of the study and consent were taken.

## Intervention:

For the treatment phase, three groups of interventions were chosen at random, each with 30 patients.

Group 1: Flap surgery following non-surgical periodontal therapy after one month with no oral administration of analgesic.

Group 2: Flap surgery following non-surgical periodontal therapy after one month with oral administration of Ketorolac 400mg one hour prior to the surgery.

Group 3: Flap surgery following non-surgical periodontal therapy after one month with oral administration of 2 mg Melatonin one hour prior to the surgery.

## Periodontal surgical procedures:

Two skilled and experienced periodontists (CK and SM) carried out all surgical procedures while adhering to defined surgical standards for the surgeries. For the first minute, all patients received additional oral antisepsis with 2% chlorhexidine. Then, local infiltrative anesthetic (2% lidocaine with epinephrine 1:100,000) was administered before the operation. There were no more than 6ml reported anesthetic solutions. The administration of long-acting medicines was avoided to avoid confusing effects. Then, the area between the central incisor and second molar was subjected to an open-flap debridement procedure using a kirkland flap technique. We used manual (Gracey curettes) and ultrasonic tools for scaling and root planing. The surgery was completed within 45 minutes post drug administration.

## Postoperative instructions and data collection:

Data regarding outcome measures, time duration, amount of anaethesia used that influenced the results of the current investigation were recorded. All patients received the same postoperative instructions at the conclusion of each procedure, including verbal and written advice about physical activity limitations, oral hygiene, and local hemostatic measures. Postoperative medications were prescribed.

## Outcomes and data collection:

Complete periodontal charting was done at baseline.

## Primary outcome:

[1] Visual analog scale - Wong-Baker faces pain scale

[2] FLACC Score

[3] Oxygen saturation level- SPO2

## Secondary outcome:

[1] Systolic blood pressure

[2] Diastolic blood pressure

[3] Heart rate

VAS and FLACC scores were taken post treatment. Systolic and diastolic blood pressure along with heart rate was taken at 3 intervals - baseline, 20 minutes into the procedure and immediately after the procedure. Side effects were registered at the end.

**Figure 1:**
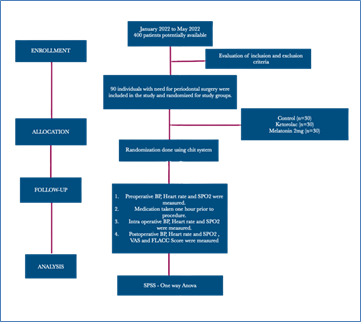
Flowchart of patient recruitment and study group randomization, according to the CONSORT statement.

Complete protocol is charted in Figure 1

## Blinding:

Participants were blinded from the start of the experiment to the end of the statistical analysis after being assigned to intervention.

## Statistical analysis:

The statistical analysis was carried out using the SPSS statistical software (Version 25.0; IBM). The information was given as a mean value with a standard deviation. The primary and secondary outcomes were compared using the Mann-Whitney U Test. The threshold for statistical significance was established at 0.05. Patients' reported side effects were expressed as absolute frequencies.

## Results:

Table 1 details the characteristics of the study sample in terms of gender and age. Gender and age were not statistically different across groups (P = 0.759 and 0.873, respectively). Table 2 displaysthe pain scores determined by the VAS scale and FLACC Score of each group. The intragroup comparisons (control-test drug) demonstrated that melatonin and ketorolac showed positive preemptive effect which compared to the control with mean differences significantly different from zero. However, when melatonin and ketorolac were compared there was no significant difference in postoperative pain among patients. After the drugs were administered, the systolic blood pressure and SpO2 briefly increased before progressively declining. During various stages of periodontal surgery, neither of the groups has significantly affected systolic blood pressure, diastolic blood pressure, oxygen saturation, or heart rate (Table 3).

## Discussion:

Non-steroidal anti-inflammatory drugs have been used for treating pain for more than a decade. However, non-selective NSAIDS act by impeding the action of cyclooxygenase enzymes, have a great negative impact such as gastrointestinal toxicity, nephrotoxicity and hematologic toxicity.[17] This current investigation aimed to fill the lacunae of avoiding the side effects of NSAIDS as preemptive analgesic and see the effect of melatonin as the same. This current research indicates that melatonin showed similar positive preemptive analgesic effect as ketorolac. In this investigation, VAS and FLACC were the primary outcome to evaluate the effect of preemptive analgesia. Melatonin and ketorolac revealed a beneficial anticipatory impact compared to the control with mean differences statistically different from zero, according to intragroup comparisons (control-test drug). However, there was no discernible difference in postoperative pain between individuals when melatonin and ketorolac were examined. No studies evaluating the effect of melatonin as preemptive analgesic for periodontal flap surgeries were found. Dexamethasone, eterocoxib, and celocoxib have all been utilized in studies on preemptive analgesia in periodontal procedures along with other steroidal and non-steroidal medications. [18,19,20] Although many theories have been put out, the precise mechanisms underlying melatonin's analgesic benefits remain unknown. Some of these theories include the involvement of -endorphins, GABA receptors, opioid l-receptors, and the nitric oxide (NO)-arginine pathway. [21] The production of -endorphin from the pituitary gland is increased by melatonin, and it has been shown that naloxone, which prevents -endorphin from binding to opioid receptors, may counteract the nociceptive effects of melatonin. [22] By interacting with opioidergic, benzodiazepinergic, muscarinic, nicotinic, serotonergic, and 1 and 2-adrenergic receptors found in the central nervous system as well as the dorsal horn of the spinal cord, melatonin may also mediate its analgesic effect. Additionally, since naloxone has the potential to counteract the long-term analgesia that melatonin induces, opioid receptors are probably implicated in melatonin function. [23] Melatonin's efficacy has been debatable, even though it has been hypothesized that it may have analgesic and calming effects during the perioperative phase. Melatonin has been used as analgesic in fibromyalgia, irritable bowel syndrome and migraine. [24] Melatonin has been proven to be effective in a number of experimental animal models of pain. [25] [26] Thus, intra peritoneal (i.p.) injection of melatonin was able to prolong the anti-nociceptive effect in models of electrically produced pain by up to 210 minutes. [27] Similarly, melatonin (120 mg/kg, i.p.) significantly reduced pain in a mouse model of thermally induced discomfort as evaluated by tail-flick. In a hot-plate model of pain induction in mice, it was found that melatonin exerted its greatest analgesic effect when given to the mice in the evening. [27,28] These effects of melatonin could also be blocked by the central benzodiazepine antagonist flurmazenil or the opiate antagonistnaloxone, suggesting that these receptor pathways interact to produce melatonin's analgesic effects. [29] Melatonin treatment exerted analgesic benefits lasting for a period of one hour in a paw-withdrawal test using a neuropathic pain model. [30] 2-Bromomelatonin was discovered to have a dose-dependent analgesic effect in a model of mechanically generated pain using tail clamping. [31] Melatonin significantly lessened chemically produced pain, which is similar to acute pain in people. Melatonin was discovered to lessen inflammation-related discomfort, most likely through inhibiting inducible NO synthase's ability to produce NO and NO-cyclic GMP's ability to signal. Melatonin was administered in all of these many animal models of pain, and it had no negative effects. Additionally, there are restrictions on melatonin's capacity to function as an anti-nociceptive substance. [32] Melatonin was able to decrease paw-withdrawal latencies, which is a measure of thermal hyperanalgesia in a mouse model of neuropathic pain in which the animals experienced a tight ligation of the sciatic nerve, but it had no discernible impact on withdrawal thresholds, a measure of mechanical allodynia. [30]^31^ [33] It is important to note several of the present study's limitations, including the sample's unique qualities, the timing of the evaluations, the scale used to evaluate pain, and certain potential spillover effects. Therefore, it is necessary to apply limitations to any generalisation of the study's findings. Preemptive analgesia has been shown to be effective in several forms of periodontal surgery,[19, 20,22,23,24,25, 26,27,28,29,30,31, ,33,34,35] however the subject is still debatable and there is no set strategy for each type of surgery. In this way, it adds more pertinent data to help clinicians decide whether to utilize preventive analgesia during open flap periodontal surgery.

## Conclusion:

The adoption of a preemptive medication protocol using either melatonin or ketorolac may be considered effective for pain and discomfort prevention during and post open-flap debridement surgeries. Melatonin showed similar effect to gold standard ketorolac in terms of its preemptive analgesic effect additional to having anti-inflammatory effect. Further studies are required to standardize the protocol for using melatonin as preemptive analgesic for dental surgical procedures.

## Figures and Tables

**Table 1:** Characteristics of study groups in relation to gender and age

VARIABLES	STUDY GROUPS			P Value
	GROUP 1 (n=30)	GROUP 2 (n=30)	GROUP 3 (n=30)	
GENDER				
MALE	18(60%)	14(47%)	17(57%)	0.759^a^
FEMALE	12(40%)	16(53%)	13(43%)	
AGE (years; mean± s.d)	40.13±3.7	41.35±4.5	40.87±4.2	0.873^b^
Group 1- Control, Group 2- Ketorolac, Group 3- Melatonin, a- Chi-squared test, b- Anova test.

**Table 2:** Postoperative pain scores of study groups

VARIABLE	GROUPS	Mean± Standard deviation	P (intragroup comparison)	P (intergroup comparison)^c^
VAS SCORE	CONTROL	3.26±0.19	0.000^a^*	0.984
	KETOROLAC	3.63±0.19		
	CONTROL	3.26±0.19	0.000^b^*	
	MELATONIN	3.70±0.19		
FLACC SCORE	CONTROL	4.01±0.37	0.000^a^*	0.677
	KETOROLAC	4.76±0.12		
	CONTROL	4.01±0.37	0.000^b^*	
	MELATONIN	4.56±0.14		
a; control-ketorolac, b; control-melatonin, c; difference between the control-test drug differences,*statistically significant.

**Table 3:** Comparison for blood pressure, peripheral capillary oxygen saturation and heart rate using ANOVA test

VARIABLE	GROUPS	SUBGROUPS		
		I	II	III
SYSTOLIC BP	CONTROL	135±6.18	132±4.44	125±3.23
	KETOROLAC	135±6.18	131±5.03	124±3.23
	p(intragroup comparison)a	0.512	0.71	0.14
	CONTROL	135±6.18	132±4.44	125±3.23
	MELATONIN	135±6.18	128±6.09	120±1.27
	p(intragroup comparison)b	0.512	0.65	0.12
	p(intergroup comparison)c	0.32	0.61	0.12
DIASTOLIC BP	CONTROL	89±3.2	85±2.8	86±1.9
	KETOROLAC	85±2.0	81±3.4	79±2.5
	p(intragroup comparison)a	0.11	0.13	0.9
	CONTROL	89±3.2	85±2.8	86±1.9
	MELATONIN	83±4.2	83±7.9	80±1.02
	p(intragroup comparison)b	0.21	0.18	0.11
	p(intergroup comparison)c	0.15	0.14	0.18
HEART RATE	CONTROL	93.6±12.1	83.1±10.3	73.6±11.3
	KETOROLAC	92.3±13.4	83.2±12.1	72.7±10.9
	p(intragroup comparison)a	0.29	0.33	0.17
	CONTROL	93.6±12.1	83.1±10.3	73.6±11.3
	MELATONIN	92.1±10.2	82.3±11.1	72.5±8.9
	p(intragroup comparison)b	0.27	0.54	0.18
	p(intergroup comparison)c	0.23	0.53	0.34
SPO_2_	CONTROL	96.3±8.5	97.7±8.3	100.3±10.1
	KETOROLAC	96.9±9.3	98.4±9.7	99.2±1.2
	p(intragroup comparison)a	1.1	0.96	0.16
	CONTROL	96.3±8.5	97.7±8.3	100.3±10.1
	MELATONIN	97.4±8.7	98.9±9.1	99.3±2.2
	p(intragroup comparison)b	1.12	0.56	0.23
	p(intergroup comparison)c	1.3	0.54	0.45
a; control-ketorolac, b; control-melatonin, c; difference between the control-test drug differences.
